# Community perceptions and attitudes on malaria case management and the role of community health workers

**DOI:** 10.1186/s12936-017-1916-7

**Published:** 2017-07-04

**Authors:** Collins J. Owek, Elizabeth Oluoch, Juddy Wachira, Benson Estambale, Yaw A. Afrane

**Affiliations:** 1 Afya Bora Consortium Fellowship Programme, Global Health Leadership, College of Health Sciences, University of Nairobi, Nairobi, Kenya; 2 Mild May International, Kisumu, Kenya; 3 Department of Behavioral Sciences, School of Medicine, Moi University, Eldoret, Kenya; 4 Jaramogi Oginga Odinga University of Science and Technology, Bondo, Kenya; 5 Department of Medical Microbiology, College of Health Sciences, University of Ghana, Accra, Ghana

**Keywords:** Perception, Attitudes, CHWs, Community Case Management of malaria (CCMm)

## Abstract

**Background:**

Community Case Management of malaria (CCMm) is one of the new approaches adopted by the World Health Organization for malaria endemic countries to reduce the burden of malaria for vulnerable populations. It is based on the evidence that well-trained and supervised community health workers (CHWs) can provide prompt and adequate treatment to fever cases within 24 h to help reduce morbidity and mortality associated with malaria among under-five children. The perception and attitudes of the community members on the CHWs’ role is of greater importance for acceptance of their services. The aim of the study was to assess community’s perception and attitude towards CCMm and on CHWs who undertake it.

**Methods:**

This study was conducted in five districts in western Kenya where Community Case Management was being undertaken. This was a qualitative cross-sectional study in which in-depth interviews and focus group discussions were conducted with mothers of under-five children and key stakeholders.

**Results:**

Overall, there were more positive expressions of perceptions and attitudes of the community members towards the CCMm programme and the role of CHWs. The positive perceptions included among others; recognition and appreciation of services of CHWs, bringing health services to close proximity to the community, avoiding long queues in the health facilities, provision of health education that encourages good health practices, and promotion of positive health-seeking behaviour from within the communities. This programme is not without challenges as some of the negative perceptions expressed by the community members included the fact that some clinicians doubt the capacity of CHWs on dispensing drugs in the community, some CHWs do not keep client’s secrets and mistrust of CHWs due to conflicting information by government.

**Conclusions:**

It was evident that the community had more positive perceptions and attitudes towards the role of CHWs in CCMm than negative ones. There should however, be deliberate efforts towards sustaining the positive aspects and addressing the negative concerns raised by the community and the health care practitioners.

## Background

Malaria is a major public health problem in Africa despite the continuous intervention that has been rolled out in the past decade in many parts of the continent. In the year 2015, 429,000 deaths were recorded in the whole world with 90% of the cases from sub-Saharan Africa with majority of fatalities occurring in children under 5 years [1]. There are currently intensive control efforts to reduce the burden of the disease. Community-based Case Management of malaria (CCMm) is one of the new approaches adopted by malaria endemic countries to reduce the burden of malaria for vulnerable populations [2].

According to the World Health Organization (WHO), CCMm is based on the evidence that well-trained and supervised community health workers (CHWs) can provide prompt and adequate treatment to fever cases within 24 h to help reduce morbidity and mortality associated with malaria among under-five children in Africa [1]. The WHO defines CHWs as members of the communities where they work, who should be selected by the communities, be supported by the health system but not necessarily a part of its organization, and have shorter training than professional health workers [3]. In general terms, they receive a training programme that can be from 1 to 2 weeks to more than 3 months.

Community health workers are in level one of the Kenyan healthcare service provision and a central pillar of primary health care delivery at the community level. Furthermore, Kenya’s Community Health Strategy 2006 highlights the provision of preventive health services by CHWs and clearly provides structures that operationalize service provision at community level [4]. CHWs provide the link between the health facilities and community members and are given responsibility for health promotion, disease prevention as well as treatment of specific diseases, such as uncomplicated malaria [5]. Since the 1980s, CHW programmes have been a cornerstone of primary health care based on the Alma-Ata declaration of 1978 [6]. There have been critical evaluations published concerning the role of CHWs, highlighting successes and problems (mainly related to sustainability and maintaining quality of care) as well as their potential in assisting in health and development issues [5, 7]. CHWs have been considered as agents linked to behavioural change and as playing a key role in the extension of formal health services [8]. They can contribute towards improvement of health of communities in Africa by reducing mortalities for children under 5 years [6, 9].

The services of the CHWs are a fundamental necessity in a comprehensive programme, and in recent years, it has become a popular methodology for linking health facility based care to community intervention and other services. CHWs play a critical role in the overstrained health care system. A CHW is a volunteer who is a trusted member of and/or has an unusually close understanding of the community served. This trusting relationship enables the CHW to serve as a liaison/link/intermediary between health/social services and the community to facilitate access to services and improve the quality and competence of service delivery. CHW lead programmes are currently being scaled-up in many countries of sub-Saharan Africa to improve access to healthcare. However, the perception of the community members, who are used to dealing with the formal health system, is crucial in the implementation and success of CCMm. This study seeks to assess community’s perception and attitude towards Community Case Management of malaria and on CHWs who undertakes it.

## Methods

### Study sites

The study was conducted in five districts in western Kenya where Community Case Management was being undertaken by CHW. These consisted of three districts in the malaria endemic lowland region namely Kisumu West, Nyando and Muhoroni and two districts (Kisii and Kenyenya) in the epidemic prone highlands of western Kenya. The endemicity of malaria in the highland and lowland sites are different. The topography of highland sites consist of hills and valleys with mosquito breeding sites only located in the valleys which makes malaria transmission being focal in the valley bottoms [10]. Malaria transmission is also hypoendemic and can be epidemic in these regions [10, 11]. In lowland sites mosquito breeding sites are spatially distributed and malaria transmission is hyperendemic and perennial.

### Study subjects

The study subjects consisted of mothers/guardians of under-five children who have received services from the CHWs in the past 1 year. CHWs from community units where CCMm was being undertaken were interviewed in focus group discussions. Other study subjects were key informants that included community health extension workers (CHEWs) who supervise the CHWs, heads of health facilities in which the CHWs are attached to, clinicians within these health facilities, Public Health Officers and District and County Medical Officers of Health.

### Study design

This study was a qualitative cross-sectional study in which in-depth interviews and focus group discussions (FGDs) were conducted with the study participants using a semi-structured questionnaire. The studies were conducted in five districts with two community units (CU) where CCMm was being undertaken by CHWs chosen from each district for the study by convenience sampling. One community unit in the Kisumu West district was used to pilot the study. Questionnaire looked for information such as the perception of mothers/guardians of under-five children and other key informants on CCMm, the appropriateness and attitude of the CHWs who deliver CCMm, among others. Questions to the CHWs were to gather information about the perception of their work and attitude of their clientele towards them. These studies were undertaken from May to September 2014.

### Sample size

In Kenya, each district has been divided into community units that consist of a few villages that makes up a sub-location. Within each community unit are 10–12 CHWs depending on the population. Each CHW is assigned one hundred (100) households to work with. This consist of approximately 500 people. In each community unit, ten (10) CHWs who have been trained on CCMm were picked for the FGDs. This will represent CHWs working in 1000 households or with 5000 people per community unit, assuming the average household consists of five people. Thus, in total 100 CHWs were picked for the study, who were working in 10,000 households and with 50,000 people. Within each community unit, ten (10) mothers/guardians of children under the age of 5 years who have enjoyed the services of the CHWs, were selected for the study. In total, 100 community members were selected for the study from the 10 community units within the five districts.

From each community unit, the CHEW who supervises the CHWs within that unit was selected for key informant qualitative interviews. For each community unit, the clinician-in-charge of the health facility where the CHWs were attached or their deputy was chosen for key informant interviews. Therefore, a total of ten (10) CHEWs and ten (10) clinicians were interviewed for this study as key informants. For each of the five districts, the Public Health Officer (PHO) and the district or county director of health was also interviewed as a key informant.

### Data collection

The interview guide developed was pre-tested in a non-study area, a community unit in the Kisumu West sub-county. FGDs and key informant interviews contained questions that related to the perception of the different categories of people interviewed. These questions were varied depending on the category of person being interviewed so as to capture different themes and to answer different questions. The questionnaires for the FGDs were first written in English, translated into the local language (Dholuo or Swahili) and then back translated into English by a different person to ensure accuracy.

Mothers/caregivers of under five children from households within the same CU and who have ever been served by a CHW were brought together at the health facility to be interviewed through FGDs. These were conveniently selected from households within the study sites by our team. All CHWs from one CU were brought together at a central point in the health facility where they report, to do focus group discussions. The CHEWs were interviewed separately as key informants. The other key informants such as heads of health facilities in which the CHWs are attached to, clinicians within these health facilities, Public Health Officers in the district and District and County Medical Officers of Health were interviewed individually by semi-structured questionnaires. The questionnaire had questions on community acceptability of and attitudes towards CHWs to undertake CCMm. All study participants signed a consent form after explanation on the study purpose.

### Data management and analysis

All data collections were qualitative and were recorded by two audio recorders. These were later transcribed, translated and coded. They were finally organized into themes in relation to the objectives of the study. FGDs for CHWs and mothers from the communities were done in the local language (Dholuo and Swahili) and were later translated into English. Key informants were interviewed in English. All the transcripts were read as individual wholes to gain an overall understanding of the data and coded the transcripts using Nvivo version 7 software. A common induction approach to coding based on phenomenology allowed us to explore community perception and mothers acceptability of CHWs rather than pre-existing theory [12].

The results of this coding was compared for consistency of text segmentation and code application. When results were acceptable and consistent, the coding continued with periodic checks for continued intercoder agreement.

### Ethical considerations

The study was approved by the Scientific Steering Committee and the Ethical Review Committee of the Kenya Medical Research Institute (KEMRI) before commencement of the study. Local approval was granted by the County commissioner of Kisumu and Kisii Counties of western Kenya and all district commissioners in the selected districts. Written informed consent was obtained from all the study participants after explaining the objectives of the study in the local language to them.

## Results

### Demographic characteristics of respondents

The mothers that were interviewed in the FGDs ranged from 19 to 36 years with a median age of 24 years. The CHWs that were interviewed were aged between 25 and 59 years of age with median age of 32. CHEWs were aged between 32 and 47 years with median age of 37, whilst the ages of the clinicians and district/county director of health ranged from 28 to 52 years. Among the CHWs, 56% of them were females whilst none of the CHEWs were female. Among the clinicians and PHOs that were interviewed 40% (8/20) of them were female. Majority of the CHWs (64%) had finished secondary school with the rest having finished primary education whilst only 22% of the mothers had finished secondary school, with 54% completing primary education and 24% with no education. None of the participants declined to be part of the study.

### Positive responses

The responses from the interviews are summarized below into themes. To a large extent, most of the community members cited positive perception and attitude towards the role played by the CHWs, however, a few expressed their dissatisfaction with the role of the CHWs. In particular, when it comes to administering of anti-malarial drugs, there is a diverse view within the community and with key stakeholders. These are discussed below:

### Recognition and appreciation of the services of CHWs

Community members recognize and value the services of CHWs that they have an important role in supporting health care within the community to the point that they refer to them as “doctors”. Community members are always ready to report to the CHWs on how their children are doing health-wise. Some of the mothers reported that when they go to the hospital they are given bed nets, but are not shown how to hang it. However, CHWs are always available to help them hang the bed nets. When a pregnant woman needs assistance, they are always available to provide necessary support including referral to a nearby health facility. The community members appreciate the role that CHWs are playing in the community towards supporting sick members of the community by seeking their assistance.

*“…….I can just say thank you to the CHWs when my child is sick they do come and see how to take the child to the hospital. Even if I am not able to take him the CHW can even take care of that…, the boy was treated and he is now well*.”

 Pregnant mothers in an FGD at Kisii.

The CHWs themselves also feel that the people in their communities recognize their work and feel proud about it.

*“…. they socialize well with us, they call us to help them where they have a health problem and they also call us :doctors” and so we feel well.”*

  A CHW in an FGD at Kisii County.

*“….sometimes even when I am walking around people will just call me that why don’t you pass by and see how we are doing, so that means that they are responding positively to our services.”*

 CHW in FGD, Chemelil, Kisumu County.

*“…….they used not respect our work but as time goes by they have come to respect us, they used to say that “these are just young men that we know”, so even if you try to explain something to them, they belittle what you say but things have changed over the years…*

 CHW in FGD, Chemelil, Kisumu County.

### Bringing health services to close proximity to the community

The communities view the CHWs work on CCMm as bringing health services closer to their door step. Some communities assert that they have to walk long distances to go the health facility, with some saying they have to cross a river with a “dangerous bridge”. Therefore, if the CHWs are available to give basic health care to them then they are happy to enjoy it. According to the CHEWs, the community appreciates the role of the CHWs because they are nearer to them and especially in areas that are far from the health facilities, the people there appreciates their services.

A Public Health Officer (PHO) also shared the same view that the CHWs add value in the lives of the village communities, in testing and providing malaria drugs especially in the hard to reach areas and providing them with malaria preventing information. Another PHO was of the view that the services offered by CHWs in the communities reduces pressure on the health facilities. She asserts that in some of the health facilities in her district, more than half the people who are in a queue to see the clinician do have malaria related complaint, and that since they started CCMm in the area almost 2 years, queues to the health centres and dispensaries have reduced.

“,,,,,,*I think they should continue working, because through them many people have gone to the health center. They have referred them, and they have also enabled us to reach many people in the interior.”*

 PHO in a KII, Kenyanya Kisii County.

### Avoiding long queues in the health facility

The community members appreciate the role of the CHWs in helping them avoid long queues for testing of malaria in health facilities. According to the CHEWs, the CHWs test the people at home for malaria using rapid diagnostic test (RDTs) kits and if anyone in the community is positive, CHWs give them a referral note to go to the health facility, which they give to the clinician to be prescribed drugs. This makes many of the communities members avoid queues at the health centre to see the clinician, and then queue again when they are sent to the laboratory for malaria testing. According to a clinician in charge of a health facility, pregnant mothers feel uncomfortable sitting in a queue all day so if a CHW has already tested her at home then that helps, in that she would only be given drugs and then she goes home.

*“……..most clients prefer to be tested outside the health facility because in the facility they will queue to go to the lab to be tested so they prefer the CHWs to test them.”*

 Clinical Officer in charge of a Health facility in a KII.

### Cost of services

There was a feeling among the community members that the CHWs offer free malaria testing services and sometimes provision of malaria treatment while the health facility charge for these services. The malaria test at the health facility is supposed to be free according to the government policy. However, as of the time of the study (May–September 2014), in practice the health facilities charge the villagers. But the community members are of the view that it is better to see a CHW before going to the hospital because he will test you for free and give you a note to be taken to the health facility for treatment. In that case they avoid the fee at the health facility for the malaria test. Occasionally, the community members call the CHWs whenever a household member is sick. The CHEWs asserts that there were times that the CHWs had anti-malarial drugs that were given to their clients when they test positive for malaria with the RDTS.

### Strong linkage between the community and health facilities

The community members view the CHWs as a strong linkage between the households and the health facilities through extending health education to the households, provision of malarial drugs and referral to the health facilities when the need arises.

*“The CHWs are good because, if you have a problem they help you, they walk around and every time that they walk to your place they have to ask you about your health, how you are doing. If you tell them of a health issue, they will write to you a small note that you go with to the hospital and when you go with that note, you will be treated.”*

 Pregnant mother in an FGD at Kisumu West district.

*“When the cases are referred from the community to the health facility by the CHWs the clients actually get help, they are somehow given priority.”*

  CHEW in a KII in Kisii.

*“I think through the CHWs many people have gone to the health center through their referral system. They have enabled us to reach many people in the hard*-*to*-*reach areas.”*

 PHO in a KII in Kisumu west.

*“…..the CHWs also help during bed net distribution in the communities. They know the houses where there are under*-*fives where we have to take the nets to. They also help in other immunization campaigns.*

  A PHO in Kisii.

*“………….The CHWs helps in many areas, not to me alone but I do see them even help during polio vaccination. They assist the nurses as they know that if they come to my house there are such and such ages of the kids, they know that you have under five child and so when you refuse it will just be upon you but they have already directed the nurses to you*.”

 Pregnant mothers in an FGD at Kisii.

### Provision of health education that encourages good health practices

According to the community members, the CHWs communicates well with them and encourages them to visit health facilities. They help clients to make their individualized health plans. They conduct home visit where they provide health education, remind clients on scheduled dates with the clinic and encourage hospital delivery. They also reach the community members during mass health intervention campaigns in assisting the health workers. They educate the people on how to keep mosquitoes away from home and how to hang the bed nets at home.

*“A CHW used to visit me every week and keep reminding me about my ANC date. He also advised me to save money towards my delivery so that when the day comes I can get money for transport and to pay for any other services which is not free at the health facility.”*

 Pregnant mother in an FGD at Kisumu West.

### Confidence and trust in the CHWs

According to the CHEWs, the community members have developed trust and confidence towards the support provided by the CHWs. This confidence has grown over time. At the initial stages, some members of the communities said they had some doubts how these people “they saw growing up and not well educated” could now be giving health tips to them and testing them for malaria.

*“…..nowadays, most clients when they are not feeling well the first person they see is the CHWs and then the CHW does his or her part and then gives the necessary advice or a referral.”*

 CHEW in a KII.

The CHW perceive that they have won the confidence of their clients since many of them keep coming to them instead of going to the health facilities straight away when they are sick of malaria.

*“If you tell a client that they need to be referred to the health facility, sometimes they refuse and say just treat me here. They believe that you can completely treat them.”*

 CHW from Kisii in an FGD.

*“…they will always come for referrals, they will even come to ask me if I have paracetamol to give them and this makes me happy.”*

 CHW in an FGD at Ojolla B, Kisumu West district.

*“…..now if they see any symptoms of malaria they rush to the hospital and get tested, but before, whenever people had headache they would go to the shops and buy some drugs, at times the drugs were not for malaria but ever since we started educating them, they nowadays protect themselves from malaria and also treat it appropriately.”*

 CHWs from Kisii in an FGD.

*“In my own opinion I just think that the CHWs should just be given that full mandate to test and treat people at the community level and then the complicated cases are referred to the facility.”*

 CHEW in a KII.

### Positive health-seeking behaviour from within the communities

The community members and key informants are of the view that the role of the CHWs has contributed positively to changes in health-seeking behaviour. This has made many people to seek for prompt treatment for their children when they are have fever or perceive that they are sick of malaria.

The CHWs also feel that they have improved attitudes of health-seeking behaviour among the communities. They assert that through their referral system, many of their clients are now going to the hospitals to seek treatment early whenever they or a member of their family is sick of malaria.

*“Years ago women used to deliver at home but since we started urging them to go to the hospital, many have been coming. There are many who never used to go to the clinic when they have fever or feel they have malaria. They will rather go to buy drugs from the shop but are now going.”*

  CHW in an FGD at Kisii County.

*“Some women come to me to write them a referral letter that they will take to the hospital, they don’t just go to the hospital.”*

  CHW in an FGD at Ojolla B, Kisumu County.

*“The villagers say that since we started being CHWs, women are better placed in terms of their health, there are no home deliveries since if their time comes they definitely go to the hospital.”*

 CHW in an FGD at Ojolla B, Kisumu County.

## Negative perceptions and attitudes

### Clinicians view on CHWs dispensing drugs in the community

Some clinicians are of the view that CHWs should not be given drugs to use in the community as part of the CCMm. They think that the CHWs are not trained enough on sickness diagnosis and, therefore, not qualified to be distributing drugs. They assert that there could be misuse of the drugs by the CHWs, they might over prescribe drugs and not give the correct doses when it comes to giving ACTs to children. Also, their assertion is that sometimes the person might not be suffering from malaria alone but other ailments which the CHWs might not be in a good position to diagnose and treat. They are of the view, that CHWs could do testing of malaria in the communities and rather refer the clients to the nearest health facility to go for drugs but not give drugs to anybody.

Some of the community members still have doubts about the capacity of the CHWs to correctly test and give them and if they do have the anti-malarial drugs, to give them the correct dosage. This affects the moral of the CHWs.

*“I have heard of the challenge it was on a certain CHWs whom a certain community member thinks that she is not qualified enough to do that work. She was claiming that the lady is not qualified.”*

 PHO in a KII.

### Some CHWs do not keep client’s secrets

Some of the community members do not trust the CHWs because some of them do not keep confidential information about them.

*“….the CHW is someone that maybe you can share with her your secret and she may end up telling others.”*

  Pregnant mother in an FGD at Kisumu West district.

### Mistrust due to conflicting information by government

Sometimes the CHWs are assured that the health facilities are functional with all the basic commodities in place but when they refer a community member, they realize they cannot get commodities like anti-malarials. And in most cases when drugs are available, clients have to pay for it, yet the CHWs have communicated to the clients that the drugs are free according to the government policy. This makes them have a negative attitude towards the CHWs who refer them.

*“…at times you give someone a referral note, they go to the hospital thinking that they will be served for free, but they get there and they have to pay to be tested with their own money and when they are found positive, they are told again to go to the chemist and buy the medicine. So they find it useless because they were not given any medicine.”*

 CHWs from Kisii in an FGD at Kisii County.

Some community members believe that a CHW should always have drugs, hence they feel offended when a CHW says that he/she does not have drugs but would like to refer them to the hospital. Some CHWs also feel that they are undermined by some pronouncement from some health care workers who live in their community. They tell the villagers about their doubts on whether the CHWs are doing the right thing in the community because they never went to “school like them”.

Also in the health facility, some clinicians do not recognize the work of the CHWs, in that they will send a client who has been tested and referred by a CHW to the laboratory to be tested again for malaria. Even when the CHW accompanies the client to the health facility the client will not have any special attention from the clinicians.

*“……they will just ask you that why did you not come with the drugs to give them. Even if you explain to them why you do not have the drugs they will just think that you are lying to them.”*

 CHW in FGD, Chemelil, Kisumu County.

*“ I went with a mother who had a very sick child with fever and I had to line up with her and from that day she despised me that I made her queue when I told her we will not.”*

 CHW in an FGD at Ojolla A, Kisumu County.

*“….there is this time that I went with one of my client we sat there just to watch how the nurses were chatting, they leave the work that they are supposed to do and just keep chatting, she told me that she does not want to go back again to the hospital.”*

 CHW in an FGD at Ojolla A, Kisumu County.

*“……..In the community people do not view them the same, some have a positive attitude towards them and others definitely have the negative attitude that they see them they relate with them and they don’t think these people are really qualified to take them and to administer the drug.*”

 PHO in a KII.

## Discussion

In the present study, CCMm being undertaken by CHWs was found to be acceptable to the community members. The communities have embraced CCMm and have shown a positive attitude towards it. Even though the CHWs claim that there was initial hesitation from members of the communities, they soon identified that CCMm brings health care closer to their doorstep and helped them to avoid long queues in health facilities. They realized that diagnosis and treatment of malaria by the CHWs is actually free according to the government policy. The initial dragging of feet by communities members to accept and embrace the CHWs who undertook CCMm could be attributed to the fact that change is always difficult for human beings. Many people in the communities might be used to going to the health facility to queue to see a clinician, be tested for malaria and then be given a prescription for drugs. For this to be replaced or complimented by CHWs who are generally people they know from their communities to have less education will not be accepted overnight.

It is interesting to note in this study that members of the community will prefer to see a CHW than to go to a health facility to see a clinician. This may be occasioned by the long queues and delays encountered in health facilities and community members alleging that some clinicians are rude in the facilities, and lack of proper attention at the facilities. They would rather see someone within their village, who is closer to them that they would not have to walk long distances to, and who will test and treat them as quickly as they can go back to their homes.

CCMm might have brought health services closer to the people that they do not have to walk long distances to health facilities and avoid queuing to see a doctor or a technician in the health facility. This saves the community members a lot of time and money for transport to the health facility. It is ironical that when community members feel that they have malaria or have fever, and would visit the health facility, they would have to pay for the diagnosis of their ailment, but seeing a CHW who lives closer to their house and who would diagnose them with RDT would be free. These makes the CCMm programme to be convenient to the community members and for them to accept the CHWs who undertake it.

The success or otherwise of CCMm will depend on community acceptance of the programme, and on the CHWs who are undertaken it. The high acceptance of both in this study points to an awareness of the benefits of CCMm by the community members and the stakeholders. Concerted efforts needs to be made to address the challenges that faces this programme in order to realize the intended benefit of reducing morbidity and mortality of malaria in sub-Saharan Africa.

The acceptability of the CCMm programme in western Kenya collaborates studies from other countries that have shown that CHWs were able to correctly perform RDTs, and showed high adherence to test results [13, 14]. This proves that with proper training and supervision, CHWs could be trusted to undertake CCMm in communities in sub-Saharan Africa [13]. The fact that in different countries CHWs have also been accepted by community members to undertake CCMm [15] shows that the programme can be successful if implemented well. The benefits of CCM include prompt access to treatment to especially under five children who bear the greatest burden of the malaria disease.

The programme cannot be successful if clinicians, health facility in-charges, and technicians are not part of the implementation. There seems to be a disconnect between these categories of people in the health facilities and the CHWs who are in the communities. The fact that some of the clinicians and technicians in the health facilities shared the opinion that CHWs are not adequately trained to handle patients diagnosis and treatment in the community and are not supporting them tells the fact that they might not be well informed enough of the training of the CHWs and goal of the CCMm programme. However, if healthcare workers are part of the training of CHWs and the CHWs constantly get to be reviewed and supervised by these health care workers, they all could work in harmony to implement CCMm. For now the CHWs receive training from NGOs.

The fear of many healthcare workers needs to be addressed. For instance the clinicians fear that CHWs will misuse drugs, over treat their clients which could lead to drug resistance. CHWs might be tempted to treat everyone who presents with fever and who may test negative for malaria with the RDTs. Malaria treatment is being undertaken with powerful and expensive ACT drugs [2] and if these develop resistance in people there are no available drugs to replace them Therefore the concerns of the clinicians could be right and more training needs to be organized for the CHWs.

In conclusion, CHWs are accepted by community members in Kenya to undertake CCMm however, there are several challenges that needs to be addressed for the programme to be a success. The challenges raised here are also similar to many countries in sub-Saharan Africa. Concerted efforts from governments through the ministry of health, malaria control programmes, civil society organizations that are working on malaria prevention, health care workers and communities are needed to boost the programme to make it sustainable and to boost the morale of the CHWs to sustain and retain their services to undertake the programme.

## Abbreviations

CCMm: Community Case Management of malaria
CHWs: community health workers
CHEW: community health extension worker
PHO: Public Health Officer
FGDs: focus group discussions
RDT: rapid diagnostic test
ACT: artemether combination therapy
NGO: Non-Governmental Organization
KII: key informant interview
ANC: ante-natal care
WHO: World Health Organization
KEMRI: Kenya Medical Research Institute
